# Intersection of Epigenetic and Immune Alterations: Implications for Fetal Alcohol Spectrum Disorder and Mental Health

**DOI:** 10.3389/fnins.2021.788630

**Published:** 2021-12-03

**Authors:** Alexandre A. Lussier, Tamara S. Bodnar, Joanne Weinberg

**Affiliations:** ^1^Psychiatric and Neurodevelopmental Genetics Unit, Center for Genomic Medicine, Massachusetts General Hospital, Boston, MA, United States; ^2^Department of Psychiatry, Harvard Medical School, Boston, MA, United States; ^3^Stanley Center for Psychiatric Research, Broad Institute of MIT and Harvard, Cambridge, MA, United States; ^4^Department of Cellular and Physiological Sciences, Faculty of Medicine, The University of British Columbia, Vancouver, BC, Canada

**Keywords:** prenatal alcohol exposure (PAE), Fetal Alcohol Spectrum Disorder (FASD), development, immune function, epigenetics

## Abstract

Prenatal alcohol exposure can impact virtually all body systems, resulting in a host of structural, neurocognitive, and behavioral abnormalities. Among the adverse impacts associated with prenatal alcohol exposure are alterations in immune function, including an increased incidence of infections and alterations in immune/neuroimmune parameters that last throughout the life-course. Epigenetic patterns are also highly sensitive to prenatal alcohol exposure, with widespread alcohol-related alterations to epigenetic profiles, including changes in DNA methylation, histone modifications, and miRNA expression. Importantly, epigenetic programs are crucial for immune system development, impacting key processes such as immune cell fate, differentiation, and activation. In addition to their role in development, epigenetic mechanisms are emerging as attractive candidates for the biological embedding of environmental factors on immune function and as mediators between early-life exposures and long-term health. Here, following an overview of the impact of prenatal alcohol exposure on immune function and epigenetic patterns, we discuss the potential role for epigenetic mechanisms in reprogramming of immune function and the consequences for health and development. We highlight a range of both clinical and animal studies to provide insights into the array of immune genes impacted by alcohol-related epigenetic reprogramming. Finally, we discuss potential consequences of alcohol-related reprogramming of immune/neuroimmune functions and their effects on the increased susceptibility to mental health disorders. Overall, the collective findings from animal models and clinical studies highlight a compelling relationship between the immune system and epigenetic pathways. These findings have important implications for our understanding of the biological mechanisms underlying the long-term and multisystem effects of prenatal alcohol exposure, laying the groundwork for possible novel interventions and therapeutic strategies to treat individuals prenatally exposed to alcohol.

## Reprogramming of Physiological Systems by Prenatal Alcohol Exposure

Alcohol (ethanol) exposure *in utero* can have numerous adverse effects on a developing fetus. In humans, prenatal alcohol exposure (PAE) can result in Fetal Alcohol Spectrum Disorder (FASD), which refers to the broad spectrum of structural, neurocognitive, and behavioral abnormalities or deficits that can occur following PAE (Astley and Clarren, 2000). The magnitude of these effects is variable and depends on factors such as timing and level of maternal alcohol use, physiological and genetic background, and a host of environmental factors including overall maternal health and nutrition (Pollard, 2007). Despite the innate variability of these moderating factors, children across the entire spectrum of FASD display long-term cognitive and neurobehavioral alterations, including neurocognitive impairment (i.e., cognition, learning, memory, and executive function), impaired self-regulation (i.e., attention, impulsivity, behavioral regulation, stress responsiveness, mood/affect, and sleep), and deficits in adaptive functioning (i.e., communication, social behavior, and activities of daily living) (Streissguth and O’Malley, 2000; Astley et al., 2009; Doyle and Mattson, 2015; Lynch et al., 2015; Carter et al., 2016; Panczakiewicz et al., 2016). Taken together, these findings highlight the complex reprogramming effects of PAE on neurobehavioral, neurobiological, and physiological systems. However, the mechanisms underlying the pervasive and multisystem impacts of alcohol are not yet fully understood.

The Developmental Origins of Health and Disease (DOHaD) hypothesis provides an important framework to interpret both the transient and long-term effects of PAE (Hellemans et al., 2010). This hypothesis developed through mounting evidence indicating that early-life exposures or events can have a long-lasting impact on adult health outcomes (Barker and Osmond, 1986; Barker et al., 1989, 1993; Wadhwa et al., 2009). Importantly, the DOHaD hypothesis suggests that early environments can exert their influence on long-term health through the mechanism of fetal programming, by which early life experiences shape development of neurobiological and physiological systems, altering their function over the lifespan. However, additional research is needed to fully uncover the mechanisms that drive the reprogramming of physiological systems.

**FIGURE 1:**
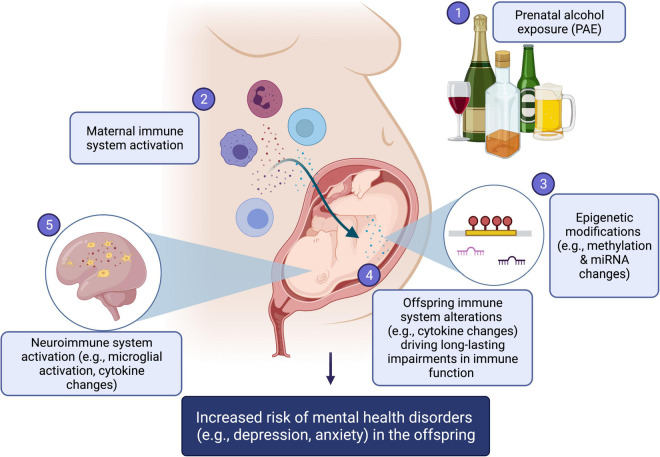
Summary figure. (1) Prenatal alcohol exposure (PAE) can result in (2) maternal immune system activation, altering the fine cytokine balance during pregnancy, which in turn impacts the developing immune system of the fetus. In turn, both direct effects of PAE and alcohol-induced maternal immune activation may result changes in (3) epigenetic mechanisms, which include alterations to DNA methylation levels, histone modification patterns, and miRNAs expression levels. These epigenetic changes are likely important mechanistic drivers of (4) life-long impairments in offspring immune function and (5) neuroimmune system alterations, including microglial activation and central cytokine changes. Together, offspring central and peripheral immune system activation, by way of epigenetic changes, are hypothesized as driving, at least in part, the increased risk of mental health conditions, such as depression and anxiety, in alcohol-exposed offspring. Created with BioRender.com.

Disruption of immune development has been recognized as a “pathway to pathology”, impacting risk for both childhood and adult diseases (Dietert, 2014). Specifically, the field of developmental immunotoxicity has identified a wide range of agents capable of immune disruption or programming of the immune system with long-term health consequences, which include diet, environmental, chemical, and physical factors such as UV radiation, and notably, alcohol/drug exposure, as well as psychological factors (Dietert, 2014). While the molecular mechanisms underlying these effects have yet to be firmly established, epigenetic mechanisms are emerging as attractive candidates for the biological embedding of environmental factors on immune function, as they may link external stimuli and physiological systems to influence health and behavior into later life (Kobor and Weinberg, 2011; Yuen et al., 2011; Shulha et al., 2013; Fujii et al., 2021). In the present review, we address the impact of PAE on immune function and epigenetic patterns, highlighting a potential role for epigenetic mechanisms in the reprogramming of immune function and risk for mental health disorders. These links are conceptually summarized in Figure 1.

## Impact of Prenatal Alcohol Exposure on Immune and Neuroimmune Function

Clinical data examining alcohol-induced alterations in immune competence in children and adults with FASD remain somewhat limited [reviewed in Bodnar and Weinberg (2013), Reid et al. (2019)]. However, one of the earliest and most consistently clinically demonstrated effects of PAE is increased infection rates. Specifically, early investigations identified that children with Fetal Alcohol Syndrome (FAS), at the more severe end of the FASD spectrum, have a higher incidence of major and minor infections, including recurrent otitis media, upper respiratory tract infections, urinary tract infections, sepsis, pneumonia, and acute gastroenteritis (Johnson et al., 1981; Ammann et al., 1982; Church and Gerkin, 1988). In addition, decreased eosinophil and neutrophil cell counts in alcohol-exposed compared to unexposed children, and decreased leukocyte response to mitogens [(Johnson et al., 1981); reviewed in Gottesfeld and Abel (1991)] were observed. Maternal alcohol consumption has also been shown to result in a 5–7-fold increase in the rates of chorioamnionitis (inflammation of the chorion, amnion, and placenta) (Rickert et al., 1998; Aly et al., 2008), which is a common cause of preterm birth (Galinsky et al., 2013), and associated with higher rates of poor neurological outcomes due to inflammation and placental perfusion defects (Pappas et al., 2014). Recently, Gauthier et al. (2004) also reported that very low birth weight newborns exposed to alcohol *in utero* have a 15-fold higher incidence of early-onset sepsis compared to matched controls. Furthermore, high levels of maternal drinking (binge drinking), specifically during the second trimester, has also been shown to increase the risk of infection by approximately 4-fold, compared to that of unexposed newborns (Gauthier et al., 2005). Similarly, Libster et al. (2015) have reported increased rates of adverse infection outcomes in young children (<2 years of age) with PAE who were hospitalized for acute respiratory infections.

Several studies have also explored the link between PAE and atopic disorders (mainly dermatitis, eczema and asthma). Whereas PAE is associated with an increased risk of dermatitis (Linneberg et al., 2004; Carson et al., 2012), the risk of eczema is less clear, as there are conflicting reports of PAE increasing risk of eczema (Wada et al., 2016) and having no associations with eczema (Apfelbacher et al., 2011; Shaheen et al., 2014). By contrast, there are consistent reports of a lack of association between PAE and asthma, with studies including a range of developmental time points and alcohol exposure levels (Oleson et al., 1998; Yuan et al., 2004; Magnus et al., 2014; Shaheen et al., 2014; Wada et al., 2016).

Although the mechanisms underlying the immune-teratogenic effects of PAE remain unclear, alcohol consumption in adulthood has been shown to increase circulating cytokine levels (Crews et al., 2006; He and Crews, 2008), with chronic alcohol consumption during pregnancy increasing levels of key cytokines in both the fetus and mother (Ahluwalia et al., 2000). Importantly, as maternal cytokine induction can have a considerable impact on fetal development (Deverman and Patterson, 2009), alcohol-induced maternal immune system activation may drive some of the adverse developmental outcomes that occur following PAE. Indeed, our work has shown that alcohol consumption during pregnancy alters the maternal cytokine milieu through activation and/or inhibition of key cytokine networks (Bodnar et al., 2018). Importantly, these distinct maternal immune profiles predict neurodevelopmental status, distinguishing children with high risk or resilience to alcohol-induced neurodevelopmental delay^1^ from typically developing children (Bodnar et al., 2018). We have also shown that child cytokine networks are themselves disrupted following PAE and that network activity patterns again differ based on neurodevelopmental status of the child (i.e., typical development vs. neurodevelopmental delay) (Bodnar et al., 2020), further linking cytokine disruptions to altered developmental outcomes. Finally, while studies investigating immune outcomes of PAE in older individuals is limited, there is evidence for increased rates of atopic conditions and elevated lymphocyte counts in adolescents with PAE (Oleson et al., 1998). However, despite clear evidence of immune alterations and associated developmental alterations following PAE, further studies are needed to elucidate the mechanism(s) underlying the impact of alcohol exposure on immune function. Importantly, *in utero* alcohol exposure appears to induce long-lasting changes in immune function; however, most immune cells themselves are not long-lived, and as such, mechanistic investigations will be required to fill this gap.

Animal model experiments have allowed for more in-depth explorations of the immune disturbances associated with PAE, and in particular, have allowed for investigations into the impact of PAE on the neuroimmune system. Work from a range of animal models has shown that alcohol exposure generally increases cytokine production within the brain, a marker of neuroimmune activation (reviewed in Table 1). In third trimester equivalent exposure models, alcohol increased cytokine levels in the cerebellum, cortex, and hippocampus (Drew et al., 2015; Topper et al., 2015). Our laboratory has identified alterations in cytokine levels in the brain following alcohol exposure throughout gestation (i.e., first and second trimester equivalent), with increased cytokine levels observed in the hippocampus, and prefrontal cortex but decreased cytokine levels observed in the hypothalamus (Bodnar et al., 2016). Despite inherent differences among the various models, such as method and timing of alcohol administration, species, and cytokine detection method, the overall concordance of these findings highlights that neuroinflammation may be a cross-cutting feature in both FASD and animal models of PAE.

**TABLE 1:** Studies showing the impact of prenatal/early postnatal (third trimester equivalent) alcohol exposure on central cytokine levels.

**References**	**Species**	**Ethanol administration model**	**Age at tissue collection**	**Sex**	**Tissue**	**Cytokine/Chemokine**	**Direction**	**Method**
Drew et al., 2015	Mouse (C57BL/6)	Intra-esophageal gavage at 4 g/kg/day from P4-9	P10	Males, females (combined)	Hippocampus	IL-1β, TNF-α, CCL2	↑	mRNA
					Cerebellum	IL-1β, TNF-α, CCL2	↑	
					Cortex	IL-1β, TNF-α	↑	
Topper et al., 2015	Rat (Sprague Dawley)	Alcohol vapor (8.03 g/dL ± 21 at 4 h) from P3-5	P4, 6	Male	Cerebellar vermis	IL-1β	↑	mRNA
			P4			TNF-α	↑	
			P4		Hippocampus	TNF-α	↑	
Bodnar et al., 2016	Rat (Sprague Dawley)	Liquid ethanol diet (6.37% v/v) from GD 1–21	P8	Female	Hippocampus	IL-1β, IL-2, TNF-α, IFN-γ, IL-4, IL-5	↑	Protein
					Prefrontal cortex	IL-6, IL-5	↑	
					Hypothalamus	IL-1β, IL-2, TNF-α	↓	
Boschen et al., 2016	Rat (Long Evans)	Intragastric intubation (5.25 g/kg/day) from P4-9	P10	Male	Hippocampus	CCL4, TGF-β	↑	mRNA
Tiwari and Chopra, 2011	Rat (Wistar)	Intragastric intubation (5 g/kg/day) from P7-9	P28	Male	Hippocampus	TNF-α, IL-1β, TGF-β	↑	Protein
					Cortex	TNF-α, IL-1β, TGF-β	↑	
Roberson et al., 2012	Mouse (C57BL/6)	Intraperitoneal administration (0.03 mL/g) on GD8	6 h after ethanol treatment	Unknown	Whole embryo	IL-6, CXCL1, G-CSF IL-1β, IL-13	↑ ↓	Protein
Zheng et al., 2014	Mouse (C57BL/6)	Intraperitoneal administration (0.03 mL/g) on GD8	E9.25	Unknown	Brain	IL-1β, TNF-α	↑	Protein
Cantacorps et al., 2017	Mouse (C57BL/6)	Water containing alcohol (20% v/v) from GD 1–21	P70	Male	Prefrontal cortex	IL-1β	↑	Protein
Terasaki and Schwarz, 2016	Rat (Sprague Dawley)	Intragastric intubation (2 g/kg twice per day) GD 10–16	E17	Female, male	Hippocampus/cortex	CCL3, CCL6, CCL9, CXCL9, CXCL11, IL-21, IL-5, TNF-β, Osm, TNF-α, TNFrsf10	↑	mRNA
				Male		CCL2, CCL5, CCL9, CXCL10, IL-5	↓	
				Female		CCL2, CCL5, CCL9, CXCL10, IL-5	↑	

*GD, gestational day; E, embryonic day; P, postnatal day.*

*Studies involving a second hit/challenge were excluded.*

In addition to alterations in cytokine levels, alcohol exposure can alter microglial levels and/or activational status (Fernandez-Lizarbe et al., 2009; Kane et al., 2011; Drew et al., 2015; Topper et al., 2015; Boschen et al., 2016; Ruggiero et al., 2018; Chastain et al., 2019; Gursky et al., 2020) [reviewed in Wilhelm and Guizzetti (2015), Mahnke et al. (2019), Kane and Drew (2021)]. During early development, microglia, resident macrophages of the CNS, exist in an activated state. In this state, microglia produce cytokines (Fujita et al., 1981; Ling and Wong, 1993) and contribute to brain development through their important roles in neurobiological processes, including phagocytosis of newborn neurons (Marin-Teva et al., 2004), synaptic pruning and maturation (Paolicelli et al., 2011), remodeling of synaptic circuits (Tremblay et al., 2011), and synaptic plasticity (Xavier et al., 2014). As a result, alterations in microglial populations and activational status may be an important mechanism through which alcohol exposure impacts early brain development. By weaning, microglia transition to a quiescent state and remain relatively inactive throughout adulthood, unless activated by injury or immune challenge (Ling and Wong, 1993). However, alcohol exposure may impair/delay the transition of microglia to a quiescent state (Drew et al., 2015) and as such, may result in heightened responses to challenges such as infection, with potential consequences for behavior and cognition [reviewed in Bilbo and Schwarz (2012)]. Thus, microglia are uniquely poised to retain an immunological memory of early-life insults, such as exposure to alcohol, due the long-lived nature of these cells amid a more ephemeral immune cell background. Nevertheless, the mechanisms underlying the impact of alcohol exposure on cytokine levels and microglial activation are not fully known. Based on evidence of fetal programming by PAE and from other models where epigenetic alterations of microglia are associated with neuroinflammation (Jovicic et al., 2013; Chauhan et al., 2015), we propose that epigenetic influences may be a critical link, tying together alcohol exposure, long-term impacts on immune function and subsequent health outcomes.

## Epigenetic Mechanisms Bridge Early-Life Environments and Long-Term Health

Epigenetics refers to modifications of DNA and/or its regulatory factors that mediate the accessibility of DNA, which can, in turn, modulate gene expression and cellular functions without changes to underlying genomic sequences (Bird, 2007). These regulatory factors include histone modifications, non-coding RNA (ncRNA), and direct DNA modifications, such as methylation and hydroxymethylation. In general, epigenetic patterns are closely associated with cellular specification and differentiation, highlighting their role in the regulation of cellular functions (Ziller et al., 2013). As each cellular subtype is closely associated with a characteristic epigenomic landscape that provides long-term stability to its identity, cell type is the main driver of stable epigenetic patterns. However, environmental stimuli can also influence epigenetic patterns throughout the genome, albeit with subtler effects than ontogenic profiles. These mechanisms rely on an apparent paradox between the stability of cell-specific profiles and plasticity in response to external cues to modulate both short- and long-term epigenetic regulation (Boyce and Kobor, 2015; Aristizabal et al., 2020). Overall, epigenetic patterns act in concert to fine-tune the cellular response to external stimuli and regulate cellular functions [reviewed in Allis and Jenuwein (2016)]. Importantly, emerging evidence suggests that epigenetic patterns, such as DNA methylation, may mediate the relationship between environmental insults and chronic disease, highlighting a potentially crucial role in our understanding of the biological embedding of early-life exposures (Fujii et al., 2021).

## Prenatal Alcohol Exposure Alters Epigenetic Programs

The initial evidence that epigenetic mechanisms might be involved in programming of physiological function by PAE originated from studies of gene expression. Genome-wide alterations to gene expression patterns occur in the brains of fetal, neonatal, and adult animals following PAE, highlighting the possibility that the effects of alcohol may shift early developmental trajectories and lead to persistent alterations in adulthood (Hard et al., 2005; Green et al., 2007; Zhou et al., 2011b). One recent example comes from a study identifying large-scale alterations to neuroimmune gene networks of the olfactory system (Gano et al., 2020). Our work has shown that PAE animals show changes in the brain’s transcriptome both under basal conditions and in response to an immune challenge, supporting the reprogramming of neuroendocrine and neuroimmune function by alcohol (Lussier et al., 2015). Studies investigating possible epigenetic mechanisms of developmental alcohol exposure followed closely behind the initial gene expression studies, identifying widespread alterations to epigenetic profiles in both central nervous system and peripheral tissues, including DNA methylation, histone modifications, and miRNA expression [reviewed in Lussier et al. (2017)]. These findings have highlighted a potential role for epigenetic factors in the reprogramming of neurobiological functions by PAE in both animal models of PAE and clinical cohorts of individuals with FASD. Although relatively few epigenome-wide studies have been performed to date, several have identified alcohol-induced alterations to genes involved in immune function (Liu et al., 2009; Zhou et al., 2011a; Khalid et al., 2014; Laufer et al., 2015; Marjonen et al., 2015; Chater-Diehl et al., 2016; Portales-Casamar et al., 2016; Frey et al., 2018; Lussier et al., 2018a,b; Sharp et al., 2018; Cobben et al., 2019; Table 2). These findings provide further evidence that the immune deficits observed following PAE may be linked to changes in epigenetic patterns.

**TABLE 2:** Differential effects of PAE on the epigenetic profiles of immune genes.

**Measure**	**References**	**MODEL**						**RESULTS**		IMMUNE GENES*						
		**Species**	**Tissue**	**Age**	**Treatment (ethanol dosage, length, timing)**			**Total genes**	**% in immune genes**	**Antigen Processing/Presentation**	**Antimicrobials**	Complement system*	Cytokines and chemokines*	Receptors*	B or T cell receptor signaling*	**Natural Killer Cell Cytotoxicity**
**DNA methylation**	Liu et al., 2009	Mouse	Embryo	GD 10	400 mg/dL	44 h	GD 8.25	279	7.9%	KLRC3, PSMC3	FABP5, HMOX1, **PDGFRA**, SLPI	**CFP**	**CRH**, CSH2, GHRH, LEFTY1, **PDGFRA**, PTHLH	FGFRL1, **IGF2R**, IL1RAP, IL4RA, TNFRSF8, **VIPR2**	MALT1, PDK1, PPP3CB	KLRC3, PPP3CB, PTPN11
	Hicks et al., 2010	Mouse	NS-5 neural stem cells (cultured)	NA	400 mg/dL + FGF2	2 days	NA	46	6.5%		JUN		DTL	**TUBB3**	JUN	
		Mouse	NS-5 neural stem cells (cultured)	NA	400 mg/dL + TGFB1	2 days	NA	146	2.7%		BIRC5			**TUBB3**	PAK1, PAK3	PAK1
	Laufer et al., 2013	Mouse	Whole brain	PN 70	10% v/v	Free drinking	GD 1 - PN 10	27	0%							
	Chater-Diehl et al., 2016	Mouse	Hippocampus	PN 70	2.5 g/kg (2× at 0 h and 2 h)	Subcut. injection	PN 4,7	38	7.9%		IL18, VDR		IL18, TAC1	VDR		
	Zhou et al., 2011a	Rat	DRG-derived neural stem cells (cultured)	NA	400 mg/dL	6 h	NA	91	12.1%		AQP9, CRABP1, OASL1, TNF		ARMET, CLCF1, INSL6, TNF		PIK3R1, PTPRC, TNF	NCR3, PIK3R1, SH3BP2, TNF
	Lussier et al., 2018a	Rat	Hypothalamus	PN 1, 7, 14, 21	Liquid ethanol diet	22 days	GD1-22	34	11.8%		**IFIH1**, TMPRSS6			CCRL2, IL20RA		
	Lussier et al., 2018a	Rat	Hypothalamus; white blood cells	PN21	Liquid ethanol diet	22 days	GD1-22	32	9.4%	AP3B1			FGF9	IL18R1		
	Krishnamoorthy et al., 2013	Human	Embryonic stem cells (cultured)	NA	20 mM	4 days	NA	0	NA							
	Khalid et al., 2014	Human	Embryonic stem cells (cultured)	NA	20 mM or 50 mM	1 or 2 days	NA	392	12.2%	MR1	CABP1, CCBP2, CCL11, CXCL2, **IFIH1**, IL12B, IL2, IL4, PGC, PGLYRP3, PMP2, PPARG, SRC, TFRC, TNFSF10	CD96, **CFP**	CCL11, CMTM6, **CRH**, **CSF1**, CSHL1, CXCL2, EREG, FGF17, FGF18, IL12B, IL2, IL23A, IL4, MSTN, NPY, PTH, TNFSF10	CCBP2, CSF3R, ESR1, **FGFR2**, HNF4A, LTBR, MCHR2, NGFR, NR1H4, NR2E3, PPARG, RORA, **TACR1**, TSHR	CARD11, IL2, IL4, SYK, ZAP70	**ITGAL**, NCR1, PRKCA, SH2D1A, SYK, TNFSF10, ZAP70
	Laufer et al., 2015	Human	Buccal epithelial cells	3–6 years	Confirmed FASD			199	5.0%			COLEC11	CMTM2, PDGFRL, **PTHLH**, SEMA4C	ESRRG, FGFR3, **VIPR2**	IFITM1, LYN	
	Portales-Casamar et al., 2016	Human	Buccal epithelial cells	5–18 years	Confirmed PAE			465	7.1%	**HLA-DPB1**	BCL3, CCR3, **DES**, GDF15, KNG1, LAP3, LRP1, NOX5, PLSCR1, RBP4, ROBO3, S100A7A, S100A8	C1R, C1S, CD47, CSMD1	CSF2, FGF21, GDF15, LHB, PROK1, UCN3	BMPR1B, CALCR, CCR3, CRHR2, ESRRB, LAP3, PTH1R, ROBO3, RXRA, **TACR1**, TNFRSF10D	CSF2, NFATC1	CSF2, **ITGAL**, NFATC1, TNFRSF10D
	Lussier et al., 2018b	Human	Buccal epithelial cells	3.5–18 years	Confirmed FASD			143	6.3%	HLA-DPA1, **HLA-DPB1**	**DES**, KLRK1		AGT, FGF12	**FGFR2**, IL1R1	AKT3	KLRK1
	Sharp et al., 2018	Human	Cord blood	Birth	Sustained drinking through pregnancy			86	5.8%	NFYA, TAPBP			**SLIT3**, TNFSF14	NR2E1		
					Binge			158	8.9%		CACYBP, CXCL12, GDA, IL29, NFKBIZ, RPF1	CFHR2	ANGPTL5, CXCL12, IL29, LTBP4, PYY, TNFRSF11B	NR2F2, ROBO2, TNFRSF1A		
					First trimester exposure			35	11.4%		**PDGFRA**		FGF6, **PDGFRA**	CXCR7, RORB		
					Second and third trimester exposure			81	4.9%	HLA-C			ATF1, **SLIT3**		IKBKB	HLA-C
	Frey et al., 2018	Human	Buccal epithelial cells	7.44 years ± 0.55	>30 ng/g ethyl glucuronide (EtG) in meconium at birth			189	7.4%	HSPA4	AHNAK, DEFB121, PENK, SRC	ITGB2	**CSF1**, PENK, SAA2, SEMA3B, **SLIT3**	MTNR1B, NRP1, SCTR	PIK3CD	ITGB2, PIK3CD
	Cobben et al., 2019	Human	Blood	1.0–18.4 years	Confirmed FASD			18	5.6%					TNFRSF19		
**H3K4me3**	Chater-Diehl et al., 2016	Mouse	Hippocampus	PN 70	2.5 g/kg (2× at 0 h and 2 h)	Subcut. injection	PN 4, 7	35	No immune genes identified in top 40 differential loci							
**H3K27me3**	Chater-Diehl et al., 2016	Mouse	Hippocampus	PN 70	2.5 g/kg (2× at 0h and 2h)	Subcut. injection	PN 4, 7	39	2.6%					S1pr2		

*Data were obtained from immport.org. Total genes represents the total number of genes associated with alcohol exposure. Bolded genes were present across multiple studies.

*The cytokines and chemokines category includes chemokines, cytokines, interleukins, TGFb family members, TNF family members, and interferons (none found).*

*The receptors category includes receptors for chemokines, cytokines, interleukins, TGFb family members, TNF family members, and interferons (none found).*

*The B or T cell signaling category includes BCR signaling pathways and TCR signaling pathway.*

## Epigenetic Programs Play a Key Role in Immune System Development and Function

Epigenetic programs are crucial to the broader development and function of the immune system, playing important roles in the regulation of immune cell development and identity as well as neuroinflammatory processes (Garden, 2013). Given the vital role of epigenetic mechanisms in the regulation of cell fate, it is perhaps not surprising that epigenetic mechanisms play an important part in the developmental cascades associated with immune cell differentiation (Obata et al., 2015). In particular, epigenetic processes regulate stem-cell properties of progenitor cells and become increasingly specialized as immune cells progress through lineage commitment. The vital importance of these patterns is exemplified by the deficits in immune development and function in animals lacking components of the epigenetic machinery, including DNA methyltransferase (DNMTs) and histone deacetylases (HDAC) (Lee et al., 2001; Akimova et al., 2012; Dovey et al., 2013; Obata et al., 2015). miRNAs also play a key role in immune system development, displaying unique expression signatures in different cellular subtypes, including microglia, granulocytes, and monocytes, which likely help modulate their specific functions and developmental trajectories (Taganov et al., 2006; Johnnidis et al., 2008; Hashimi et al., 2009; Schmidl et al., 2018). Moreover, the activation state of immune effectors relies on epigenetic mechanisms, particularly histone modifications, to induce the phenotypic alterations necessary for their rapid response to pathogens (Aung et al., 2006; Nicodeme et al., 2010; Satoh et al., 2010; Obata et al., 2015; Zhang and Cao, 2019). Of particular relevance to the current review, the epigenetic profiles of microglia are closely linked to neuroinflammatory processes, reflective of their role in the immune response of neural tissues (Jovicic et al., 2013; Chauhan et al., 2015; Kaminska et al., 2016). Importantly, the epigenetic responsivity of microglia to both external and internal signals may play a crucial role in modulating the inflammatory status of the brain, which has important ramifications for neurobiological functions (Cheray and Joseph, 2018). Overall, immune system development occurs in parallel with epigenetic changes in immune cells, which are responsive to both environmental and biological cues.

Several lines of evidence also suggest that developmental exposures can influence epigenetic patterns within the developing organism to potentially alter immune function and susceptibility to neurobiological deficits later in life (Hinz et al., 2012). For instance, animal model studies suggest that increased maternal care can alter IL-10 expression and DNA methylation in microglial cells to diminish morphine-induced addictive behavior (Schwarz et al., 2011; Wang et al., 2018). Such findings highlight the role of early life experiences in shaping developmental trajectories within the immune system and suggest that epigenetic mechanisms could play an integral role in the reprogramming of immune functions by PAE.

## Epigenetic Mechanisms May Influence the Immune Alterations Associated With Prenatal Alcohol Exposure

Studies investigating epigenetic mechanisms involved in PAE effects have also identified alterations to cytokines, chemokines, and signaling pathways involved in the cellular response to immune molecules. Table 2 outlines the findings from genome-wide studies of PAE, highlighting epigenetic alterations to genes involved in immune response and regulation (immune gene annotations obtained from immport.org, May 2021) (Bhattacharya et al., 2018). Of particular note, results from work on a cohort of children with FASD reported that DNA methylation levels in buccal epithelial cells showed alterations in HLA-DPB1, a component of the major histocompatibility complex previously associated with rheumatoid arthritis (Raychaudhuri et al., 2012; Liu et al., 2013; Portales-Casamar et al., 2016; Lussier et al., 2018b). Importantly, both evidence from animal models (Zhang X. et al., 2012) and reports from a recent informal health survey in adults with FASD (Himmelreich et al., 2020) indicate that the incidence of rheumatoid arthritis is higher following PAE. While the findings of altered HLA-DPB1 were identified in a peripheral tissue not involved in immune modulation, they may provide insight into changes in global epigenetic patterns associated with altered immune profiles in children with FASD. Members of the complement system, a key immune pathway that promotes inflammatory responses to combat infection (Sarma and Ward, 2011), also appear across multiple studies, from animal models to clinical cohorts of individuals with FASD. For instance, CFP displays alterations in both mouse and human embryonic cells exposed to alcohol, while C1R and C1S show alterations in the peripheral tissue of individuals with FASD (Khalid et al., 2014; Portales-Casamar et al., 2016).

Through the use of animal models, where one can assess the brain directly, several candidate genes involved in the reprogramming of neuroimmune functions have been identified as being altered following PAE, particularly genes within the TNF receptor (TNFRSF1A, TNFRSF8, TNFRSF10D, TNFRSF11B, and TNFRSF19) and CXC/CC chemokine receptor families (CCR3, CCRL2, and CXCR7) (Liu et al., 2009; Khalid et al., 2014; Portales-Casamar et al., 2016). Several studies also reported alterations to epigenetic patterns in members of the TNF (TNF, TNFSF10, and TNFSF14) and CXC/CC chemokine (CCBP2, CCL11, CXCL2, and CXCL12) families themselves (Table 2). Together, almost every study of epigenetic patterns following PAE identified alterations to genes involved in cytokine/chemokine/interleukin expression and signaling, showing clear parallels and links to changes in circulating levels of these markers described earlier. Importantly, several immune system genes were common across model organisms and human populations, including CFP (Liu et al., 2009; Khalid et al., 2014), CRH7 (Liu et al., 2009; Hellemans et al., 2010), CSF1 (Khalid et al., 2014; Frey et al., 2018), FGFR2 (Khalid et al., 2014; Lussier et al., 2018b), ITGAL (Khalid et al., 2014; Portales-Casamar et al., 2016), PGFRA (Liu et al., 2009; Sharp et al., 2018), PTHLH (Liu et al., 2009; Laufer et al., 2015), and VIPR2 (Liu et al., 2009; Laufer et al., 2015), which may point to potential pathways for mechanistic follow-up studies to assess whether they may be suitable targets for intervention.

Beyond genes directly involved in immune system functioning, several immune-related transcription factors also show differential epigenetic profiles following PAE and could play a role in the altered genomic response to immune signals. Of note, PPARG (PPAR-γ), a transcription factor that promotes anti-inflammatory processes (Le Menn and Neels, 2018), shows differential DNA methylation and expression in the brain following PAE and has been previously implicated in the prevention of alcohol-induced cell death (Kane et al., 2011; Khalid et al., 2014). The polycomb group proteins are also altered by PAE and have been implicated in the deficits caused by PAE, as they play a key role in modulating the properties of neural stem cells and immune cell progenitors (Aloia et al., 2013; Veazey et al., 2013). Taken together, epigenetic alterations to immune genes provides a potential mechanistic link between PAE and long-lasting immune system dysfunction.

In addition to DNA and protein-based epigenetic alterations, several differentially expressed miRNAs known to be critical regulators of neuroimmune function have also been identified in PAE models, including miR-9, -21, -153, -155, and -335 (Sathyan et al., 2007; Wang et al., 2009; Balaraman et al., 2012; Jovicic et al., 2013; Ignacio et al., 2014; Qi et al., 2014). For example, alcohol exposure increases miR-155 expression, which typically promotes the secretion of pro-inflammatory cytokines by microglial cells following Toll-like receptor activation (Cardoso et al., 2012; Ignacio et al., 2014). By contrast, alcohol decreases miR-21 expression, a neuroprotectant that suppresses Fas ligand levels, which may lead to greater vulnerability to microglial-induced cell death (Sathyan et al., 2007; Balaraman et al., 2012; Zhang L. et al., 2012). These data suggest that developmental alcohol exposure may shift the balance of different neuroimmune cell types in the brain, as well as the cytokines they produce, setting the stage for more robust neuroinflammatory responses. This possibility represents an important consideration for epigenetic studies, as cell type proportions are the major drivers of epigenetic patterns and must be taken into consideration when analyzing these types of data. To this point, a recent single-cell RNA-sequencing study of GD14.5 mice exposed to binge-levels of alcohol showed that PAE altered the cell cycle status of microglia in the ventricular zone, suggesting that alcohol may shift the developmental trajectories of neuroimmune pathways and mechanisms (Salem et al., 2021). These results also highlight the importance of timing in the study of PAE, as identifying the developmental trajectories of key neurobiological pathways may provide profound insight into the mechanisms that drive the effects of PAE on neurodevelopmental and physiological outcomes. Taken together, these findings suggest a complex interplay between the immune system and epigenomic profiles, which may, at least partially, influence the neurobiological and neuroinflammatory profiles observed following PAE, and, in the future, may enable us to describe unique immune and neuroinflammatory signatures in FASD.

## Epigenetic Dysregulation of Immune Function – The Missing Link Between Prenatal Alcohol Exposure and Mental Health Disorders?

Individuals with FASD experience high rates of mental health problems. In the general population, approximately 20% of individuals experience a mental health disorder (Nestler et al., 2002), whereas 90% of individuals with FASD have a mental health disorder, with anxiety and depression among the most common (Famy et al., 1998; O’Connor et al., 2002; Pei et al., 2011). Although the molecular mechanisms underlying this increased vulnerability in alcohol-exposed individuals remains unclear, alterations in the epigenetic regulation of immune genes resulting in abnormal immune/neuroimmune functioning has been implicated in the pathophysiology of a number of mental health disorders (Alam et al., 2017). For instance, individuals diagnosed with major depressive disorder (MDD) show increases in circulating leukocytes and proinflammatory cytokine production [reviewed in Hodes et al. (2015)], with higher childhood levels of IL-6 and C-reactive protein (CRP) potentially predating the onset of depression (Khandaker et al., 2014). Importantly, these differences are linked to epigenetic alterations, as blood cells from individuals with a lifetime history of depression show alterations to DNA methylation in IL-6 and CRP (Uddin et al., 2011). Beyond these gene-specific epigenetic alterations, recent evidence from human studies shows that epigenetic risk scores for higher inflammatory status, measured through CRP levels, are associated with increased internalizing and externalizing behaviors in children (Barker et al., 2018).

Taken together, these findings suggest a correlation between alterations in immune function and increased risk of mental health disorders, which may be mediated, at least in part, through epigenetic alterations. While this connection has yet to be specifically evaluated following PAE, the high prevalence of mental disorders in individuals with FASD concomitant with lasting alterations to immune function and epigenetic programs highlight a need for future mechanistic studies that explore this complex bidirectional relationship.

## Conclusion and Future Directions

As a whole, it is becoming increasingly apparent that a multisystem approach is needed to gain a better understand of mechanisms underlying the teratogenic effects of alcohol. To that end, we propose that immune disturbances arising as a result of *in utero* alcohol exposure may have long-term consequences extending beyond direct immune functions (e.g., protection from pathogens) to include an impact on mental health and that this may be occurring through the mechanism of epigenetics. However, the findings from epigenetic studies must be interpreted with caution, as the vast majority are correlative rather than causative in nature. As such, they do not provide a direct link between molecular mechanisms and disease and further studies are needed prior to making inferences as to causality. Nevertheless, the findings from epigenetic studies have provided important insights into potential regulatory mechanisms of immune reprogramming and may represent future targets to investigate the molecular underpinnings of alcohol-induced deficits.

Interactions between the gut microbiome, immune system, and brain are now emerging as potential moderators of neural function and potentially disease, although their connection to neuroepigenetics and neuroinflammation remain mostly unknown (Alenghat et al., 2013; Stilling et al., 2014; Carabotti et al., 2015; Petra et al., 2015; Mohajeri et al., 2018; Morais et al., 2021). Moving forward, and with this multisystem approach in mind, it will be important that future research also consider the impact of PAE on the gut-brain-immune axis (Louwies et al., 2019), as to date, there is no research in this area. It is, however, known that chronic alcohol consumption results in compromised gut-barrier function and increased rates of dysbiosis (Keshavarzian et al., 2001; Bull-Otterson et al., 2013) and as a result, *in utero* alcohol exposure would be expected to have an impact on the immature, developing gut. Moreover, dysbiosis during early life is linked to a proinflammatory state and an increased incidence of inflammatory-related diseases in adulthood (Yan et al., 2011; Hooper et al., 2012; Tamburini et al., 2016). Alterations in the microbiome may also confer increased risk of disease by altering immune system development and potentially inducing long-term epigenetic changes in immune regulators (Tamburini et al., 2016; Alam et al., 2017). Importantly, the establishment of the gut microbiome appears to rely partially on epigenetic mechanisms to establish microbe - T-cell mutualism, suggesting a complex interplay between physiological systems to dynamically regulate interactions between the microbiome and immune system (Obata et al., 2015). Thus, in the context of the present overview, future work to investigate the impact of *in utero* alcohol exposure on the gut microbiome and the gut-brain-immune axis will complement the growing body of work on immune and epigenetic alterations in preclinical PAE models and clinical studies of individuals with FASD.

Finally, a better understanding of mechanisms underlying the teratogenic effects of PAE will also pave the way for the development of more informed, targeted intervention strategies for individuals with FASD. Unlike other neurodevelopmental disorders where the underlying cause(s) are still under investigation, such as autism spectrum disorder (Won et al., 2013) or schizophrenia (Fatemi and Folsom, 2009), alcohol is a known teratogen and intervention is the key to better long-term outcomes. Due to the pervasiveness of immune disturbances across PAE models (Drew et al., 2015; Topper et al., 2015), and the link between immune function and overall physical and mental health (Raison et al., 2006), the immune system may be an ideal pharmacological target for individuals with FASD. Moreover, immune activation/cytokines play a key role in brain development, and increasing evidence demonstrates that altered immune activation may underlie altered cognition, attention, behavior, self-regulation, and adaptive functioning. Thus research on immune-based interventions will have broad implications for improving overall function of individuals with FASD [reviewed in Drew and Kane (2014)]. As such, future investigations examining the safety and utility of anti-inflammatory agents applied during early postnatal life will be important. This is particularly urgent in that currently, with the exception of ongoing work to evaluate the therapeutic potential of choline^2^ supplementation (Thomas et al., 2000, 2007; Wozniak et al., 2015, 2020; Nguyen et al., 2016) and evaluation of pioglitazone^3^ in animal models (Kane et al., 2011; Drew et al., 2015) there are relatively few available drugs specifically shown to significantly improve the outcomes of PAE.

As a whole, the collective findings from animal models and clinical studies of FASD point to a compelling relationship between the immune system and epigenetic pathways, which may have important causal links to the long-term and multisystem effects of PAE. Ultimately, additional research in this area will not only provide deeper insight into the molecular mechanisms that influence mental health processes, but also help identify novel interventions and therapeutic strategies that may alleviate the adverse health consequences arising from alcohol exposure.

## Author’s Note

Data for Table 2 were obtained from https://www.immport.org/shared/genelists.

## Author Contributions

AL and TB conceptualized and wrote the manuscript. All authors contributed to manuscript revision and read and approved the submitted version.

## Conflict of Interest

The authors declare that the research was conducted in the absence of any commercial or financial relationships that could be construed as a potential conflict of interest.

## Publisher’s Note

All claims expressed in this article are solely those of the authors and do not necessarily represent those of their affiliated organizations, or those of the publisher, the editors and the reviewers. Any product that may be evaluated in this article, or claim that may be made by its manufacturer, is not guaranteed or endorsed by the publisher.

## Footnotes

1 Neurodevelopment was assessed using the Bayley Scales of Infant Development, Second Edition (BSID-II) administered at 6 and 12 months. Children were considered to have neurodevelopmental delay if they received a score of <85 on either the Mental Development Index (MDI) or the Psychomotor Development Index (PDI) at 6 and/or 12 months.

3 Pioglitazone is a PPAR-γ agonist and PPAR-γ agonists have been shown to prevent ethanol-induced neuronal and microglial loss, as well as dampen microglial activation.
